# Supplementary material for: Resolving the full spectrum of human genome variation using Linked-Reads
Source: Genome Res. 2019 Apr;29(4):635–45. doi: 10.1101/gr.234443.118 (PMC6442396; doi:10.1101/gr.234443.118)
Supplement: Supplemental Material [file supp_gr.234443.118_Supplemental_longranger-2.2.zip › longranger-2.2.tar.bz2!/lr2.2-public/lib/stan/cmdstan/stan/logos/stanlogo-main.pdf]

STAN
